# Menopausal hormone therapy and comprehensive postmenopausal care in gynecologic cancer survivors: A position paper from the FIGO Committee on Women at Menopausal Age

**DOI:** 10.1002/ijgo.71188

**Published:** 2026-07-22

**Authors:** Agnaldo Lopes da Silva‐Filho, Suvarna Khadilkar, Hema Divakar, Chiara Benedetto, Andrea R. Genazzani, Diana Ramos, Elizabete Argale, Enrique Herrera, Martha Hickey

**Affiliations:** ^1^ Department of Gynecology and Obstetrics, Faculty of Medicine Federal University of Minas Gerais (UFMG) Belo Horizonte Brazil; ^2^ FIGO Committee on Women at Menopausal Age FIGO London UK; ^3^ Gynecology and Obstetrics Department Bombay Hospital Institute of Medical Sciences Mumbai India; ^4^ Bombay Hospital Medical Research Center Mumbai India; ^5^ FIGO Division of Well Woman HealthCare FIGO London UK; ^6^ Divakar Specialty Hospital Bengaluru Karnataka India; ^7^ Sant'Anna University Hospital Torino Italy; ^8^ Division of Obstetrics and Gynecology, Department of Clinical and Experimental Medicine University of Pisa Pisa Italy; ^9^ California Surgeon General Sacramento California USA; ^10^ Keck University of Southern California School of Medicine California USA; ^11^ Women's Preventive Services Initiative Implementation Committee American College of Obstetricians and Gynecologists (ACOG) Washington D.C Maryland USA; ^12^ Department of Obstetrics and Gynecology Riga Stradins University Riga Latvia; ^13^ Department of Obstetrics and Gynecology; Coordinator, Area of Endocrinology, Gynecology and Human Reproduction Universidad del Valle Cali Colombia; ^14^ Department of Obstetrics, Gynaecology and Newborn Health University of Melbourne Melbourne Victoria Australia

**Keywords:** cancer survivors, female genital neoplasms, genitourinary syndrome of menopause, hormone replacement therapy, hot flashes, premature menopause, quality of life, treatment‐induced menopause

## Abstract

Recent advances in cancer screening, diagnosis, and treatment have greatly improved survival rates among women with gynecologic cancers. More survivors now live long enough to experience treatment‐related menopause. Vasomotor symptoms, genitourinary syndrome of menopause, sexual dysfunction, sleep issues, and long‐term risks from estrogen deficiency can seriously impact quality of life. Despite increased survival, managing menopausal symptoms remains difficult, as concerns about cancer recurrence and hormone therapy safety often result in inconsistent or insufficient treatment. This article explores clinical considerations for menopausal hormone therapy (MHT) in women with a history of gynecologic cancer. It reviews evidence on the safety of MHT after vulvar, vaginal, cervical, uterine, and ovarian cancers, providing an overview of tumor histology, stage, and hormonal sensitivity. It also synthesizes observational studies, systematic reviews, and international guidelines to help clinicians deliver menopausal care to cancer survivors. Current evidence suggests that systemic MHT may be suitable for carefully chosen survivors of non–hormone‐dependent gynecologic cancers, including most vulvar, vaginal, cervical, and epithelial ovarian cancers. Patients with hormonally sensitive tumors should generally avoid systemic MHT, while local vaginal hormonal administration and non‐hormonal options remain important in these cases. Given the disproportionate burden of gynecologic cancers in low‐ and middle‐income countries, where younger women face increased risks of treatment‐induced menopause and limited survivorship resources, this paper emphasizes context‐specific, resource‐aware strategies to promote equitable care. By enhancing personalized and evidence‐based decision‐making, we aim to improve quality of life for women during menopause after cancer treatment, while ensuring safe, fair, and widely applicable survivorship approaches.

## 1 INTRODUCTION

Cancer remains a major challenge to global public health, with approximately 19–20 million new cases diagnosed each year. Women account for nearly half of these cases, with over 9 million new cancers diagnosed annually.^1^, ^2^ Advances in cancer prevention, early detection, surgery, systemic therapies, and supportive care have significantly improved survival rates in recent decades, resulting in a growing number of women living beyond cancer treatment.^2^, ^3^ This demographic shift has prompted a re‐evaluation of oncologic care, emphasizing the importance of cancer survivorship, which addresses the physical, psychological, and social effects of cancer and its treatment throughout the lifespan.^4^, ^5^

Survivorship care not only involves monitoring for disease recurrence but also aims to reduce the long‐term effects of treatment, improve quality of life, and promote overall health in individuals who have completed primary cancer therapy. A key aspect of this care for women treated for gynecologic cancers is managing menopausal symptoms. The management of menopause after cancer is increasingly recognized as a vital part of survivorship care.^5^

Many women are diagnosed with cancer before reaching the average age of natural menopause, especially in low‐ and middle‐income countries (LMICs) where gynecologic cancers are common.^1^ As a result, many women undergo gonadotoxic treatments, such as chemotherapy, pelvic radiotherapy, and bilateral oophorectomy, that can cause premature ovarian insufficiency or treatment‐induced menopause.^6^, ^7^

Research shows that 40%–60% of women treated for gynecologic cancers experience premature menopause. These changes are often linked to more severe vasomotor and genitourinary symptoms than those seen in natural menopause.^7^ Treatment‐induced menopause, which occurs suddenly and at a younger age, is associated with a higher frequency and greater severity of symptoms, such as hot flashes, sleep disturbances, sexual dysfunction, vaginal dryness, and mood changes, ultimately impacting quality of life.^7^

Additional reports suggest that premature ovarian failure may lead to serious long‐term health effects, including faster bone loss and higher cardiovascular risk. These conditions are affected by the postmenopausal hormonal state and by aging. This underscores the need for effective management strategies for survivorship care in women who have undergone cancer treatment.^8^, ^9^ Treatment‐induced menopause in gynecologic cancer survivors is linked to a wide range of symptoms and long‐term health effects that substantially impact quality of life and may raise morbidity. The most common symptoms and their estimated prevalence are summarized in Table 1.

**TABLE 1:** Menopausal symptoms and long‐term health effects of treatment‐induced menopause in gynecologic cancer survivors.^a^

Domain	Clinical manifestation	Estimated prevalence in cancer survivors	Clinical impact
Vasomotor	Hot flushes, night sweats	60%–80%	Sleep disruption, reduced QOL
Genitourinary	Vaginal dryness, dyspareunia, urinary urgency	40%–70%	Sexual dysfunction, GSM
Sexual health	Decreased libido, arousal disorders, anorgasmia	40%–70%	Relationship impairment, psychological distress
Psychological	Anxiety, depression, fear of recurrence, mood changes	Up to 50%	Reduced adherence to cancer treatment, impaired coping
Sleep	Insomnia, poor sleep quality	Up to 50%	Fatigue, cognitive impairment
Musculoskeletal	Arthralgia, myalgia	Variable	Reduced physical function
Bone health	Accelerated bone loss, osteopenia, osteoporosis	Increased risk with premature menopause	Fracture risk, disability
Cardiovascular	Dyslipidemia, endothelial dysfunction	Increased risk with premature menopause	Elevated cardiovascular morbidity and mortality
Cognitive	Memory complaints, brain fog	Variable	Reduced work capacity, QOL

Abbreviations: GSM, genitourinary syndrome of menopause; QOL, quality of life.

^a^ This table summarizes the most common symptoms and long‐term health effects associated with treatment‐induced menopause in gynecologic cancer survivors, with estimated prevalence based on available observational and cohort studies. Prevalence ranges are approximate and may vary according to cancer type, treatment modality, and patient age. Cardiovascular and bone health effects are influenced by both the postmenopausal hormonal environment and chronological aging.

*Source*: Hickey et al., *Lancet* 2024;^7^ Frunowicz et al., *CA Cancer J Clin* 2016;^10^ Mishra et al., *Lancet* 2024.^9^

Despite its clinical importance, the recognition and treatment of menopause after cancer remain inconsistent across survivorship programs worldwide. Many healthcare providers mainly focus on cancer surveillance, neglecting the management of menopausal symptoms and related long‐term health issues. This gap is even more prominent in LMICs that face limited access to specialized menopause care and systemic healthcare barriers.^11^, ^12^

MHT is considered the most effective treatment for managing vasomotor symptoms and genitourinary syndrome of menopause (GSM) in the general population, improving quality of life, sleep, and bone health.^7^ However, its use in women with a history of cancer remains controversial due to safety concerns. These worries often result in significant undertreatment of survivors experiencing severe menopausal symptoms, despite increasing evidence supporting the safe use of hormone therapy in selected groups, especially those with non–hormone‐dependent cancers.^12^, ^13^ This may be a different risk assessment, with only local vaginal estrogen treatment. Effective risk and benefit communication is vital for shared decision‐making. Clinicians should use a structured discussion framework that covers type of MHT (combined‐progestin vs estrogen‐only), route of administration (transdermal vs oral), regimen (sequential vs continuous), dose, and duration.^14^ This depends on whether the patient has undergone a hysterectomy, since in that case only estrogen is needed. However, in the presence of the uterus, protective progestin supplementation is clearly needed. When discussing harms and benefits, absolute risk per 1000 women over specific periods is preferable to relative risks, especially considering safety concerns after cancer. In addition, only local vaginal estrogen administration can be effective in the treatment of vaginal dryness and reduced dyspareunia with reduced risks as compared with systemic hormone replacement therapy.

## 2 SEARCH STRATEGY AND SELECTION CRITERIA

This position paper's recommendations are based on a narrative review of published literature and an evaluation of professional guidelines. It is not a systematic review but a clinical position statement created by an expert committee to synthesize current evidence and offer practical guidance for clinicians. We searched MEDLINE (via PubMed), Embase, the Cochrane Library, and Google Scholar from inception to March 2026, using keywords and MeSH terms tailored to each section. Search terms included combinations such as “gynecologic cancer,” “cervical cancer,” “endometrial cancer,” “ovarian cancer,” “vulvar cancer,” “vaginal cancer,” “uterine sarcoma,” “cancer survivor*,” “menopause,” “premature ovarian insufficiency,” “treatment‐induced menopause,” “menopausal hormone therapy,” “hormone replacement therapy,” “vasomotor symptom*,” “hot flush*,” “hot flash*,” “genitourinary syndrome of menopause,” “vaginal dryness,” “sexual dysfunction,” “non‐hormonal treatment*,” “cognitive behavioral therapy,” “neurokinin receptor antagonist,” “fezolinetant,” “osteoporosis,” “bone health,” “cardiovascular risk,” “fertility preservation,” “ovarian transposition,” “survivorship,” “quality of life,” “low and middle income country,” and “multidisciplinary care.” These were combined with study‐design filters such as “systematic review*,” “meta‐analysis,” “randomized controlled trial*,” “clinical guideline*,” “consensus statement*,” and “position statement*.” We also reviewed websites and guidelines from relevant organizations, including the European Menopause and Andropause Society, International Gynecologic Cancer Society, National Comprehensive Cancer Network (NCCN), European Society of Gynecological Oncology, American Society of Clinical Oncology, Menopause Society, International Menopause Society, British Menopause Society, and National Institute for Health and Care Excellence (NICE). Prioritizing the most recent evidence, we focused on randomized controlled trials (RCTs), systematic reviews, meta‐analyses, and Cochrane reviews, supplemented by observational studies and expert consensus when higher‐level evidence was unavailable. Evidence strength was graded using the Oxford Centre for Evidence‐Based Medicine (OCEBM) system, with level 1 indicating systematic reviews of RCTs, level 2 representing RCTs or meta‐analyses, level 3 representing cohort or observational studies, level 4 representing case series or expert consensus, and level 5 representing expert opinion. Risks of bias were carefully evaluated, with emphasis on study design flaws, early trial termination, selection bias, and confounding factors; these are noted where relevant. Only studies published in English were included. Reference lists of selected studies and guidelines were manually checked for additional relevant publications. Given the nature of a position paper, formal, systematic quality‐assessment tools such as ROBINS‐I or the Cochrane Risk of Bias tool were not used; instead, the quality and limitations of the available evidence are discussed throughout to support our recommendations. The final recommendations were developed through iterative discussions among members of the FIGO Committee on Women at Menopausal Age. Draft sections were circulated among committee members, and consensus was reached through a structured review and agreement process.

## 3 EVIDENCE SUMMARY AND PRACTICE RECOMMENDATIONS

### 3.1 How common is treatment‐induced menopause among cancer survivors?

Treatment‐induced menopause is common among cancer survivors, especially in premenopausal women who undergo gonadotoxic therapies. Chemotherapy, pelvic radiotherapy, and oophorectomy often lead to premature ovarian failure or early menopause. Up to 80% of premenopausal women experience amenorrhea within the first year after treatment, although ovarian function may recover in some younger women.^7^

Pelvic and ovarian radiation almost always cause permanent ovarian failure, while chemotherapy‐related ovarian dysfunction is more variable and can sometimes be reversible. Cranial irradiation may also damage hypothalamic–pituitary function, leading to secondary hypogonadism.^7^

Premature menopause before the age of 40 years is less common but still significantly more frequent than in the general population, especially among women exposed to high‐dose alkylating agents, ovarian irradiation, or stem cell transplants.^6^ When menstrual recovery happens, it usually occurs within the first 2 years after treatment; permanent menopause is more likely with increasing age at diagnosis, higher total chemotherapy doses, and pelvic radiation.^7^, ^15^ As cancer survival rates continue to improve, the number of women living with the long‐term effects of treatment‐induced menopause is expected to grow.^7^

### 3.2 Ovarian preservation and fertility counseling

Fertility counseling should be provided as early as possible to all reproductive‐aged women undergoing potentially gonadotoxic cancer treatment, ideally at diagnosis and before starting therapy.^16^, ^17^, ^18^ Early referral to reproductive medicine specialists is strongly advised to ensure timely discussion of fertility preservation and ovarian function–sparing options.^16^, ^17^

Ovarian preservation and fertility‐sparing approaches may be considered for carefully selected patients with early‐stage gynecologic cancers, including cervical cancer, low‐risk endometrial cancer, and germ cell tumors, especially when pelvic radiotherapy can be avoided.^6^, ^19^ In these cases, ovarian function can be maintained in a significant number of women, particularly at younger ages.^6^ Ovarian transposition before pelvic irradiation is advised for reproductive‐aged patients when radiotherapy is planned, although success rates vary and ovarian failure can still happen due to scatter radiation or remigration.^7^, ^16^, ^17^, ^18^

Integrating fertility counseling and ovarian preservation strategies into multidisciplinary cancer care is crucial to address both reproductive potential and long‐term endocrine health in cancer survivors.^7^

#### 3.2.1 Practice recommendation

All premenopausal women receiving potentially gonadotoxic cancer treatment should undergo pre‐treatment counseling on menopause risk, fertility preservation, and long‐term hormonal effects.

### 3.3 What are the main menopausal symptoms after gonadotoxic cancer treatment?

Menopausal symptoms are very common among cancer survivors who have undergone gonadotoxic treatments.^20^ The most frequently reported symptoms include hot flushes, night sweats, vaginal dryness, sleep disturbances, anxiety, and sexual dysfunction. Vasomotor symptoms affect about 60%–80% of women after cancer treatment, while genitourinary symptoms and sexual dysfunction occur in 40%–70%, and clinically significant sleep or mood issues are reported in up to 50% of survivors.^7^

A key clinical distinction should be made between depressive symptoms related to the menopausal transition, which do not fulfill the diagnostic criteria for a depressive disorder per ICD‐11 or DSM‐5, and clinical depression.^14^ MHT may be used to help improve low mood associated with other menopausal symptoms, but it is not appropriate as a treatment for clinical depression. Women who meet the diagnostic criteria for a depressive episode should be managed following standard depression guidelines while also addressing menopausal symptoms.^21^ This distinction is particularly important for gynecologic cancer survivors, as they may experience both cancer‐related psychological issues and menopausal mood symptoms.

Compared to natural menopause, treatment‐induced menopause is usually more sudden and severe, due to the rapid drop in estrogen after chemotherapy, ovarian suppression, or pelvic radiotherapy. The symptom burden may also be worsened by adjuvant endocrine therapies, especially aromatase inhibitors and ovarian suppression with GnRH agonists, which are linked to increased vasomotor symptoms, sexual problems, and lower quality of life.^7^, ^10^

The high prevalence and severity of menopausal symptoms significantly impair quality of life and may lead to decreased adherence to long‐term cancer treatments, emphasizing the importance of systematic symptom evaluation and proactive supportive care within survivorship programs.^7^

Cognitive behavioral therapy (CBT) customized for menopause effectively treats vasomotor symptoms, sleep problems, and low mood.^14^, ^21^, ^22^ It can be provided in face‐to‐face sessions, remotely, on an individual basis, in groups, or through guided self‐help. The primary advantage is alleviating the impact of vasomotor symptoms on daily life, which is particularly beneficial for cancer survivors whose symptoms might be intensified by cancer‐related stress.

Although osteoporosis is not a menopausal symptom per se, the premature loss of ovarian hormonal function significantly increases the risk of bone loss and fractures in cancer survivors. In women without estrogen‐dependent cancers, MHT is generally advised until the average age of natural menopause to reduce bone loss. When hormone therapy is contraindicated, maintaining bone health requires targeted management, including bisphosphonates and other anti‐resorptive, bone‐sparing agents, along with sufficient calcium intake of 600–1200 mg/day and vitamin D levels above 30 ng/mL. This is evaluated through regular bone mineral density assessments using dual‐energy X‐ray absorptiometry (DXA), performed every 1–5 years based on baseline bone status and individual risk factors.^23^, ^24^, ^25^

Menopause accelerates the decline in both bone mineral density and skeletal muscle mass. When osteoporosis and sarcopenia occur together, this condition, known as osteosarcopenia, significantly raises the risk of falls, fractures, and functional impairment.^26^ Preventive measures should include resistance training and sufficient protein intake, alongside pharmacological treatments. Although MHT lowers fracture risk during use, this advantage ends after stopping, and since osteoporotic fractures mainly happen in older age, long‐term use, necessary for lasting benefit, must be balanced against cumulative risks.^14^ Therefore, MHT is not recommended as a first‐line treatment solely for osteoporosis prevention in this group.

To promote structured, patient‐centered communication and systematic symptom assessment, the use of standardized clinical tools can be helpful in routine practice. A practical menopause consultation template, created to guide clinician–patient discussions across vasomotor, psychological, sexual, and urogenital areas, is included as Table S1. Such tools may improve communication, reduce patient anxiety, and support shared decision‐making in cancer survivorship care.

#### 3.3.1 Practice recommendation

Routine assessment of menopausal symptoms using validated tools (e.g., Menopause Rating Scale [MRS] or Menopause Quality of Life [MENQOL]), should be incorporated into cancer survivorship care, alongside regular evaluation of bone health in women with premature or treatment‐induced menopause to distinguish between menopausal depressive symptoms and clinical depression, as management differs. Menopause‐specific CBT can treat vasomotor symptoms, low mood, and sleep issues, either with MHT or as an alternative if MHT is contraindicated or declined.^22^

### 3.4 What are the challenges in diagnosing menopause after cancer?

Diagnosing menopause after cancer treatment is difficult because menstrual status, symptoms, and permanent ovarian failure often do not align.^20^ Temporary ovarian suppression caused by chemotherapy or ongoing endocrine therapies, such as GnRH agonists or tamoxifen, can lead to amenorrhea and menopausal symptoms that may later resolve, especially in younger women.^7^, ^27^ Therefore, amenorrhea alone is not a reliable indicator of permanent menopause in this context.

Hormonal assays have notable limitations. Follicle‐stimulating hormone (FSH) levels can fluctuate significantly and may remain elevated during temporary ovarian suppression. FSH levels above 25 IU/L measured twice after 4 weeks indicates permanent ovarian failure. Although anti‐Müllerian hormone (AMH) reflects ovarian reserve, it does not reliably predict permanent menopause or future ovarian recovery after cancer treatment.^7^, ^8^

In addition, side effects of cancer treatment regimens can mimic or worsen menopausal symptoms, including vasomotor issues, fatigue, sleep disturbances, and mood changes, which further complicates clinical interpretation and weakens the link between symptoms and actual ovarian failure.^7^, ^28^, ^29^

Diagnosis should therefore depend on a comprehensive clinical assessment, combining treatment history, long‐term symptom monitoring, and careful interpretation of hormonal measurements when appropriate. No universally accepted diagnostic standards exist, and personalized follow‐up remains crucial.^7^, ^9^

#### 3.4.1 Practice recommendation

Diagnosis should depend on a thorough clinical assessment, including treatment history, systematic symptom tracking, and long‐term follow‐up. In women aged under 45 years, persistent amenorrhea lasting more than 12 months after cancer treatment may suggest menopause unless ovarian function returns. An FSH level above 25 IU/L can also strongly indicate ovarian failure.

### 3.5 When is MHT appropriate after gynecologic cancer?

MHT remains the most effective treatment for vasomotor symptoms and GSM. In women who experience menopause after cancer treatment, however, the decision to start MHT requires careful assessment of tumor type, hormone sensitivity, stage, and the overall oncologic context.^7^ The timing of MHT initiation after cancer treatment remains poorly defined. Most studies indicate that hormone therapy can be considered once primary cancer treatment has been completed and there is no evidence of disease recurrence, especially in women with severe menopausal symptoms and low‐risk tumor biology.^7^, ^12^

MHT is indicated for the management of menopausal symptoms and should not be prescribed for the primary prevention of cardiovascular disease or dementia.^14^ The NICE 2024 guideline shows no significant subgroup differences to support the timing hypothesis for cardiovascular benefit. No trial has proven MHT prevents or delays cognitive decline or dementia. Cardiovascular risk factors during menopause should be managed with standard strategies such as lifestyle changes, blood pressure control, and lipid‐lowering therapy.

In general, systemic MHT is considered acceptable for survivors of non–hormone‐dependent cancers, while it is contraindicated or should be used cautiously in hormone‐dependent malignancies, especially estrogen receptor–positive breast cancer and certain gynecologic tumors.^7^, ^13^, ^20^ The decision to add progestins needs to be evaluated based on whether the uterus has been removed. Local vaginal estrogen therapy is usually deemed appropriate for treating GSM, even in women with contraindications to systemic MHT, because of its minimal systemic absorption. Its use should still be personalized and, when suitable, discussed within a multidisciplinary team.^7^, ^30^ The timing of initiation of local vaginal estrogen may also influence clinical outcomes. Evidence from estrogen receptor biology suggests that prolonged estrogen deprivation leads to progressive loss of estrogen receptors in vulvovaginal tissues, reducing responsiveness to subsequent hormonal therapy and potentially resulting in irreversible atrophic changes.^31^ This supports early consideration of local vaginal estrogen in cancer survivors with genitourinary symptoms.

When MHT is prescribed for cancer survivors, treatment should follow the same principles used in the general population, including using the lowest effective dose and regularly reassessing risks and benefits. Transdermal formulations are often preferred because of their lower thromboembolic risk profile. In women who experience premature or early menopause due to cancer treatment, MHT may be recommended until the average age of natural menopause, provided there are no contraindications.^7^, ^30^ The decision to prescribe MHT after gynecologic cancer should be based on tumor histology, hormonal sensitivity, disease stage, and the strength of available evidence. Table 2 summarizes the current evidence and recommendations for each gynecologic cancer type, broken down by level of evidence according to the OCEBM grading system.

**TABLE 2:** Hormonal sensitivity, MHT eligibility, and level of evidence by gynecologic cancer type.^a^

Cancer type/subtype	Hormonal sensitivity	Systemic MHT	Local vaginal estrogen	LoE (OCEBM)	Key considerations
Vulvar cancer (SCC)	No	Acceptable	Acceptable	3	Not estrogen‐dependent; individualized care; local measures important after RT
Vaginal cancer	No	Acceptable	Acceptable	3	Limited data; no increased recurrence reported; local therapy especially useful after RT
Cervical cancer (SCC)	No	Acceptable	Acceptable	2	Strong evidence supports safety; NCCN recommends consideration of MHT after treatment
Cervical cancer (adenocarcinoma)	Low/uncertain	Use with caution	Acceptable	3	Limited data; no confirmed adverse oncologic outcomes; ET may be preferred
Endometrial cancer stage I–II, grade 1–2, endometrioid	Low–moderate	May be considered	Acceptable	2	RCT (GOG) and meta‐analyses support safety in selected patients; individualized decision
Endometrial cancer stage III–IV, grade 3, non‐endometrioid	High	Contraindicated	Use with caution	4	Aggressive histology; insufficient safety data; avoid systemic MHT
LG‐ESS	High	Contraindicated	Contraindicated	4	Highly hormone‐dependent; hormonal suppression is part of treatment
uLMS	Variable (~30% ER/PR+)	Generally avoid	Use with caution	4	Conflicting data; MDT discussion and receptor status assessment required
HG‐ESS/undifferentiated sarcoma	Low/inconsistent	Avoid	Use with caution	5	Lack of safety data; MHT generally not recommended
Uterine adenosarcoma (ER/PR+)	Moderate	Generally avoid	Use with caution	4	May respond to hormones; expert consensus advises avoidance
Epithelial ovarian cancer (HG serous, mucinous, clear cell)	Low	Acceptable	Acceptable	2	Cochrane review and meta‐analyses support safety; possible survival benefit reported
LGSOC	High	Contraindicated	Use with caution	3	Hormonally responsive; endocrine therapy used as treatment
GCT/Sex cord–stromal tumors	High	Contraindicated	Use with caution	4	Hormonally active neoplasms; estrogen may stimulate growth
BOT (serous)	Moderate	Use with caution	Acceptable	3	Caution warranted, especially with invasive implants; SDM essential
BOT (mucinous)	Low	May be considered	Acceptable	3	Less hormone‐influenced; may be acceptable in selected symptomatic patients

Abbreviations: BOT, borderline ovarian tumor; ER, estrogen receptor; ET, estrogen‐only therapy; GCT, granulosa cell tumor; GOG, Gynecologic Oncology Group; HG, high‐grade; HG‐ESS, high‐grade endometrial stromal sarcoma; LG‐ESS, low‐grade endometrial stromal sarcoma; LGSOC, low‐grade serous ovarian carcinoma; LoE, level of evidence; MDT, multidisciplinary team; MHT, menopausal hormone therapy; NCCN, National Comprehensive Cancer Network; OCEBM, Oxford Centre for Evidence‐Based Medicine; PR, progesterone receptor; RCT, randomized controlled trial; RT, radiotherapy; SCC, squamous cell carcinoma; SDM, shared decision‐making; uLMS, uterine leiomyosarcoma.

^a^ This table presents the hormonal sensitivity, systemic MHT and local vaginal estrogen eligibility, and quality of supporting evidence for each gynecologic cancer type and subtype. Level of evidence is graded according to the OCEBM system: level 1 = systematic review of RCTs; level 2 = RCT or meta‐analysis; level 3 = cohort/observational studies; level 4 = case series or expert consensus; level 5 = expert opinion. Recommendations should be individualized based on tumor biology, patient symptoms, and multidisciplinary input. “Acceptable” indicates no evidence of increased oncologic risk; “Use with caution” indicates limited data requiring individualized assessment; “Contraindicated” indicates hormone‐dependent biology with potential for harm. For BRCA1/2 or Lynch syndrome carriers undergoing premenopausal risk‐reducing surgery, international consensus strongly recommends systemic MHT until the average age of natural menopause (~51 years). Non‐hormonal alternatives should be prioritized when there is a personal history of hormone receptor–positive breast cancer.

*Source*: Hickey et al., *Lancet* 2024;^7^ Rees et al., *Maturitas* 2020;^32^ Luzarraga Aznar et al., *Int J Gynecol Cancer* 2025;^13^ Villa et al., *J Clin Med* 2024;^12^ Saeaib et al., *Cochrane* 2020;^33^ Barakat et al., *J Clin Oncol* 2006;^34^ Nebgen et al., *BJOG* 2023.^35^

#### 3.5.1 Vulvar and vaginal cancers

MHT is also considered acceptable after treatment for vulvar and vaginal cancers. These malignancies are not hormone‐dependent, and available evidence does not suggest an increased risk of recurrence associated with hormone therapy.^13^, ^32^

Most vulvar cancers are squamous cell carcinomas, which are not sensitive to estrogen. Therefore, both systemic and topical MHT may be considered for managing menopausal symptoms in vulvar cancer survivors.^36^, ^37^ Similarly, evidence suggests there is no contraindication to systemic or local hormone therapy in vaginal cancer survivors.^32^ Clinical management should, however, remain individualized. In addition to systemic therapy, local measures such as vaginal moisturizers, lubricants, and dilator therapy may be especially important for women who have received pelvic radiotherapy.^7^

#### 3.5.2 Cervical cancer

Menopause after cervical cancer often results from treatment, especially after hysterectomy followed by pelvic radiotherapy, chemoradiotherapy, or bilateral oophorectomy. Symptoms can be more severe than in natural menopause. MHT is generally considered safe after treatment for cervical cancer, particularly in survivors of squamous cell carcinoma. Evidence for adenocarcinoma is more limited but does not indicate an increased risk of recurrence after surgery.^13^, ^32^, ^38^ Current international guidelines also support this approach. The NCCN specifically recommends considering MHT for women who experience treatment‐related menopause after cervical cancer treatment.^39^

Most cervical cancers are squamous cell carcinomas, which are not dependent on estrogen. Therefore, systemic MHT is not contraindicated in survivors of squamous cell carcinoma.^40^, ^41^ A comprehensive review of hormone therapy in cervical cancer survivors found no evidence that MHT negatively impacts cancer outcomes, and some observational studies even indicate a decreased risk of cervical squamous carcinoma among women on MHT.^38^

Although cervical adenocarcinoma has been considered theoretically more hormone‐responsive than squamous carcinoma, available evidence indicates that MHT is not contraindicated in survivors of adenocarcinoma.^12^, ^42^ Some experts recommend cautious use of estrogen‐only therapy in this context, although no convincing evidence demonstrates adverse oncologic outcomes associated with its use.^43^

MHT should be considered for cervical cancer survivors who develop treatment‐induced menopause, especially those with significant vasomotor symptoms, GSM, or who are at risk for long‐term complications of estrogen deficiency such as osteoporosis or cardiovascular disease.^7^, ^39^

#### 3.5.3 Uterine cancer

##### 3.5.3.1 Endometrial cancer

The use of MHT after endometrial cancer is more complex than in other gynecologic cancers and requires careful, individualized assessment. Endometrial carcinoma is traditionally viewed as a hormone‐dependent tumor, and concerns about estrogen exposure and the potential stimulation of residual malignant cells have historically limited hormone therapy use in these patients. However, increasing evidence indicates that the oncologic safety of MHT largely depends on tumor histology, stage, and individual patient factors.

In general, MHT may be considered for carefully selected women with early‐stage, low‐grade endometrioid endometrial carcinoma who have completed definitive surgical treatment, typically involving total hysterectomy with bilateral salpingo‐oophorectomy, and show no evidence of residual or recurrent disease.^12^, ^32^ These patients usually present with stage I disease and favorable tumor biology, and their risk of recurrence after treatment is relatively low.

Evidence from randomized and observational studies indicates that MHT does not significantly raise the risk of recurrence in carefully selected patients. The Gynecologic Oncology Group conducted the only RCT on this subject, comparing estrogen therapy to a placebo in women who had prior surgery for early‐stage endometrial cancer. The study found no statistically significant difference in recurrence rates or overall survival between the two groups.^44^ In this trial, tumor recurrence occurred in 2.3% of women receiving estrogen therapy, compared to 1.9% in the placebo group, suggesting no measurable increase in oncologic risk with estrogen use in this specific population. However, the trial was terminated early due to slow participant enrollment, which limited its statistical power to detect small differences in recurrence risk.^34^

Subsequent observational studies and pooled analyses have further confirmed the safety of MHT in this context. Multiple meta‐analyses of endometrial cancer survivor cohorts have consistently found no significant increase in recurrence or mortality among women using MHT compared to non‐users.^45^, ^46^, ^47^ Some analyses even suggest a possible reduction in recurrence risk for women on hormone therapy, though these results should be interpreted with caution due to potential selection bias and confounding factors.^45^, ^46^ Taken together, these data indicate that MHT may be worth considering for women with stage I–II, grade 1–2 endometrioid carcinoma, especially when severe menopausal symptoms significantly affect quality of life. In these cases, the potential benefits of symptom relief, better sleep, improved sexual function, and enhanced overall quality of life should be weighed against the theoretical oncologic risks, which appear to be low in this carefully selected group.

In contrast, MHT remains contraindicated or strongly discouraged in patients with high‐risk endometrial cancer. This includes women with advanced‐stage disease (FIGO stage III–IV), high‐grade tumors (grade 3), and non‐endometrioid histologic subtypes such as serous carcinoma, clear cell carcinoma, and carcinosarcoma, which are known for more aggressive tumor biology and limited evidence on the safety of hormone exposure.^13^, ^48^ MHT should also be avoided in hormonally responsive uterine malignancies, such as endometrial stromal sarcoma, where estrogen stimulation might theoretically promote tumor growth.

Therefore, decisions about using MHT after endometrial cancer should be personalized and ideally involve clinicians experienced in gynecologic oncology. Important factors to consider include tumor stage and histology, presence of high‐risk pathological features, time since cancer treatment, severity of menopausal symptoms, and patient preferences.^7^ These decisions should be made within the broader context of survivorship care, which aims not only to monitor for disease recurrence but also to address long‐term treatment‐related symptoms and improve quality of life for cancer survivors.^49^, ^50^

##### 3.5.3.2 Uterine sarcomas

The use of MHT after uterine sarcomas is generally contraindicated or strongly discouraged, as several sarcoma subtypes demonstrate hormone sensitivity. Low‐grade endometrial stromal sarcoma (LG‐ESS) is a highly hormone‐dependent tumor characterized by frequent estrogen and progesterone receptor expression. Hormonal suppression using progestins, aromatase inhibitors, or gonadotropin‐releasing hormone analogs is commonly employed as part of treatment. Consequently, systemic estrogen therapy should be avoided in women with a history of LG‐ESS.^32^, ^51^ Similarly, uterine adenosarcoma, especially when hormone receptor–positive, may respond to hormones. For this reason, most experts advise against using MHT in these patients whenever possible.^51^

The role of MHT in uterine leiomyosarcoma (uLMS) remains debated. Approximately one‐third of leiomyosarcomas show estrogen and progesterone receptor positivity, and some reports indicate that hormonal exposure might promote tumor growth. Although definitive evidence is scarce, most guidelines advise against systemic MHT or recommend cautious use, preferably after a multidisciplinary evaluation and assessment of tumor receptor status.^32^, ^48^ In high‐grade endometrial stromal sarcoma and undifferentiated uterine sarcoma, hormone receptor expression is less consistent, and these tumors are usually managed with systemic therapy. Due to limited safety data, MHT should generally be avoided in these cases.

#### 3.5.4 Ovarian cancer

The suitability of MHT after surgical removal of ovarian cancer and the presence or absence of metastasis mainly depends on tumor histology, grade, and biological behavior. Overall, existing evidence suggests that MHT may be an option for many women treated for epithelial ovarian cancer, although there are notable exceptions, and treatment decisions should be personalized.^7^, ^13^ Some observational studies have even indicated improved overall survival among ovarian cancer survivors receiving MHT, although this finding should be interpreted cautiously due to potential selection bias.^52^

##### 3.5.4.1 High‐grade epithelial ovarian cancers

Most ovarian cancers are high‐grade serous carcinomas, which are not strongly dependent on estrogen. Evidence from RCTs and meta‐analyses indicates that MHT does not raise recurrence risk and may even improve survival outcomes in some groups of ovarian cancer survivors.^7^, ^33^ A Cochrane review that included RCTs found no evidence of increased recurrence and suggested a possible survival benefit for women receiving MHT after epithelial ovarian cancer treatment.^33^ Observational studies and meta‐analyses similarly show that postoperative MHT does not appear to negatively affect overall or disease‐free survival in epithelial ovarian cancer survivors.^12^, ^52^ Therefore, MHT may be considered for women with epithelial ovarian cancer who experience symptoms, especially those with high‐grade serous, mucinous, or clear‐cell tumors, after a thorough individual risk assessment and multidisciplinary discussion.^7^, ^12^

##### 3.5.4.2 Low‐grade serous carcinoma and hormone‐dependent tumors

In contrast, low‐grade serous ovarian carcinoma is a hormonally responsive tumor often treated with endocrine therapy, such as aromatase inhibitors, as part of the cancer management plan. Due to its sensitivity to estrogen, MHT is generally contraindicated in this subtype.^32^, ^48^ Likewise, granulosa cell tumors and other sex cord–stromal tumors are hormonally active neoplasms where estrogen stimulation may encourage tumor growth. Therefore, systemic MHT should typically be avoided in these patients, and non‐hormonal options are preferred.^13^, ^32^

##### 3.5.4.3 Borderline ovarian tumors

MHT in borderline ovarian tumors (BOTs) requires particular caution because the available evidence remains limited and the biological behavior of these tumors varies according to histologic subtype.^12^, ^53^ Borderline tumors represent a heterogeneous group of epithelial neoplasms characterized by atypical proliferation without stromal invasion. Although their prognosis is generally favorable, hormonal influences may play a role in certain subtypes, particularly serous tumors.^54^

Epidemiological research indicates that MHT could be linked to a higher risk of developing serous borderline tumors in the general population, especially with extended use of unopposed estrogen therapy.^55^, ^56^ However, there are limited data specifically on the risk of recurrence after BOT treatment. Some observational studies have not shown a connection between MHT and survival rates in women previously treated for BOTs.^57^ Despite this, expert consensus advises caution, particularly for women with serous borderline tumors or invasive implants.^12^, ^32^ Conversely, mucinous borderline tumors appear less affected by hormonal influences, and current data do not support a clear association with MHT.^55^ Consequently, MHT may be more acceptable for carefully chosen patients with mucinous BOTs, especially when menopausal symptoms significantly reduce quality of life.

When considering MHT after BOT treatment, decisions should consider tumor subtype, surgical management (including fertility‐sparing procedures), symptom severity, patient age, and patient preferences. Shared decision‐making and thorough follow‐up are recommended in these cases.^12^

#### 3.5.5 Considerations for hereditary cancer predisposition syndromes

Managing menopausal symptoms in women with germline pathogenic variants in cancer susceptibility genes poses unique clinical challenges. Carriers of BRCA1 and BRCA2 mutations face lifetime cumulative breast cancer risks of approximately 72% and 69%, and ovarian cancer risks of 44% and 17%, respectively.^58^ Risk‐reducing bilateral salpingo‐oophorectomy (RRBSO) remains the most effective strategy to reduce ovarian cancer mortality, with reported risk reductions in the range of 80%–95%.^59^ Current NCCN guidelines recommend RRBSO at 35–40 years for BRCA1 carriers and at 40–45 years for BRCA2 carriers, or upon completion of childbearing.^60^ However, premenopausal oophorectomy inevitably triggers abrupt surgical menopause, with well‐documented consequences, including severe vasomotor symptoms, accelerated bone loss, unfavorable cardiometabolic changes, and increased risk of cognitive decline.

A 2023 international Delphi consensus involving 27 experts from 12 countries, informed by a scoping review of over 6700 publications, strongly recommends systemic MHT after premenopausal RRBSO until at least the average age of natural menopause (approximately 51 years), both for symptom relief and to mitigate the adverse cardiometabolic and skeletal consequences associated with premature estrogen deprivation.^35^ Transdermal estradiol is preferred in women with elevated thrombotic risk, and micronized progesterone is generally favored over synthetic progestins when endometrial protection is needed.

A critical concern for patients and clinicians is whether exogenous hormones might offset the breast cancer risk reduction achieved with RRBSO. Available evidence is broadly reassuring for short‐ to medium‐term use. A prospective cohort study of 872 BRCA carriers found that estrogen‐only MHT, used for an average of 3.9 years, did not raise breast cancer risk (hazard ratio [HR] 0.97; 95% confidence interval [CI] 0.62–1.52).^61^ Similarly, another study found no significant increase in breast cancer risk with MHT use of up to 4.3 years after RRBSO, although data beyond 5 years remain limited.^62^ Most studies have evaluated estrogen‐only regimens; evidence on combined estrogen‐progestogen therapy in this population is considerably more limited, and the choice of regimen, route, and duration warrants individualized discussion.

Among women with Lynch syndrome undergoing prophylactic hysterectomy for early‐stage endometrial cancer, available meta‐analytic data suggest that estrogen‐only therapy does not increase recurrence risk (relative risk [RR] 0.53; 95% CI 0.30–0.96), with a trend toward improved outcomes among MHT users.^46^ Nonetheless, prospective evidence specific to Lynch syndrome carriers remains limited.

For all women with hereditary cancer predisposition syndromes, MHT decisions should involve a multidisciplinary team comprising gynecologic oncology, medical genetics, and menopause specialists. Non‐hormonal alternatives, including CBT, selective serotonin reuptake inhibitors (SSRIs)/serotonin‐norepinephrine reuptake inhibitor (SNRIs), gabapentin, and vaginal moisturizers, should be offered when MHT is contraindicated or declined, particularly for women with a personal history of hormone receptor–positive breast cancer.^35^

#### 3.5.6 Breast cancer risk assessment in MHT decision‐making

In clinical practice, prescribing MHT to gynecologic cancer survivors involves considering their breast cancer risk. Many of these women share risk factors such as obesity, nulliparity, genetic predisposition, and previous pelvic radiotherapy with scatter, all of which raise their baseline breast cancer risk. However, breast cancer risk is often described vaguely or qualitatively rather than being precisely quantified. This approach can result in unnecessary avoidance of MHT or inadequate informed consent.

Several validated models support individualized risk estimation. The Tyrer‐Cuzick model (IBIS tool, version 8) incorporates detailed family history, hormonal and reproductive factors, breast density, and, when available, polygenic risk scores, providing both 10‐year and lifetime risk estimates.^63^ BOADICEA (implemented in the CanRisk online platform) is particularly suited for women with known or suspected pathogenic variants because it integrates rare high‐ and moderate‐penetrance genes alongside common single nucleotide polymorphisms (SNPs) and non‐genetic risk factors.^64^ The Gail model, although widely used in primary care screening programs, has more limited applicability in this population because it excludes paternal family history and second‐degree relatives.

Updated NICE guidance (CG164, 2024 revision) defines moderate risk as a lifetime probability in the range of 17%–30% and high risk as 30% or higher, with corresponding recommendations for enhanced surveillance, genetic referral, and consideration of risk‐reducing strategies.^65^ Importantly, the NICE menopause guideline (NG23, 2024 update) explicitly states that a family history of breast cancer is not a contraindication to MHT, provided that risk has been individually assessed and the woman has been counseled accordingly.^66^ The 2022 North American Menopause Society position statement reinforces this principle, emphasizing that decisions should reflect the balance between the cardiovascular, skeletal, and cognitive harms of estrogen deprivation on the one hand and the magnitude of individual breast cancer risk on the other.^50^

We suggest including a formal breast cancer risk assessment with a validated tool as a standard step before prescribing MHT to all gynecologic cancer survivors. The results should be recorded and discussed during shared decision‐making. Women at high risk should be managed through a multidisciplinary breast, gynecology, and genetics pathway, allowing for careful consideration of MHT benefits and alternatives, alongside intensified breast monitoring protocols.

#### 3.5.7 MHT in the context of modern systemic oncologic therapies

Immune checkpoint inhibitors (ICIs), poly(ADP‐ribose) polymerase inhibitors (PARPi), and antibody‐drug conjugates (ADCs) are now established as standard parts of gynecologic cancer treatment, and they also have a direct impact on managing menopausal symptoms.

ICIs (pembrolizumab, dostarlimab, nivolumab) may cause endocrine immune‐related adverse events, most commonly thyroid dysfunction and, rarely, hypophysitis or primary ovarian insufficiency, which can mimic or exacerbate menopausal symptoms.^67^ These must be ruled out before symptoms are attributed to menopause. No clinical evidence indicates that exogenous estrogen impairs ICI efficacy, although preclinical data on estrogen‐immune modulation remain inconclusive.^68^

PARPi (olaparib, niraparib, rucaparib) are often prescribed as maintenance therapy for BRCA1/2 carriers who are in iatrogenic menopause after risk‐reducing salpingo‐oophorectomy.^69^ Their side effects, fatigue, arthralgias, myelosuppression, closely resemble menopausal symptoms, which may lead to under‐recognition. No pharmacokinetic interactions with MHT have been identified, so their co‐administration is not contraindicated; however, PARPi‐induced thrombocytopenia could heighten bleeding risk in patients receiving combined estrogen‐progestogen therapy.^70^

ADCs, such as mirvetuximab soravtansine for platinum‐resistant ovarian cancer, have not been evaluated for interactions with MHT.^71^

Importantly, no RCT has examined how MHT interacts with these modern agents. Until such data exist, decisions regarding MHT for patients on or recently finished with ICIs, PARPi, or ADCs should be made through multidisciplinary consultation and personalized risk–benefit evaluation.

#### 3.5.8 Practical implementation summary

At the point of care, clinicians should follow six sequential steps. First, confirm oncologic eligibility by checking the histologic type, disease‐free status, and agreement from the treating oncologist. Tumors such as low‐grade serous ovarian carcinoma, adult granulosa cell tumors, and low‐grade endometrial stromal sarcoma require a detailed multidisciplinary discussion before starting MHT. Second, assess breast cancer risk with a validated model (such as Tyrer‐Cuzick, CanRisk, or Gail). Patients with a lifetime risk of 30% or higher or known BRCA1/2 mutations need a multidisciplinary review; BRCA carriers who have had risk‐reducing salpingo‐oophorectomy should be strongly encouraged to use MHT until the median age of natural menopause. Third, conduct a structured symptom evaluation covering vasomotor symptoms, GSM, mood, sleep, sexual function, and musculoskeletal issues. For patients on ICIs, confirm that endocrine immune‐related adverse events are absent before attributing symptoms to estrogen deprivation. Fourth, choose the appropriate regimen: transdermal 17β‐estradiol (25–50 μg/day) with micronized progesterone (200 mg/day cyclically or 100 mg/day continuously) when the uterus is present, estradiol alone after hysterectomy, or low‐dose vaginal estrogen for localized genitourinary symptoms. In resource‐limited settings, oral estradiol valerate with oral micronized progesterone is a suitable alternative. Fifth, plan follow‐ups: at 3 months (to assess symptom response, tolerability, and bleeding) and annually (to review symptoms, perform oncologic surveillance, and reassess breast cancer risk). MHT continuation should be re‐evaluated at age 51 or after 5 years. Sixth, for patients on systemic oncologic therapies such as ICIs, PARPi, or ADCs, obtain multidisciplinary approval and monitor for specific treatment‐related complications like thrombocytopenia‐related bleeding with combined estrogen‐progestogen therapy during PARPi use. Document shared decision‐making at each visit.

#### 3.5.9 Practice recommendation

MHT after gynecologic cancer should be determined by tumor histology and hormonal sensitivity. MHT may be considered for symptomatic survivors of non–hormone dependent cancers, including most vulvar, vaginal, cervical, endometrial, and high‐grade epithelial ovarian and tubal cancers, after individualized counseling. Conversely, hormone‐sensitive tumors such as low‐grade serous ovarian carcinoma and endometrial stromal sarcoma generally contraindicate systemic MHT, and non‐hormonal therapies should be preferred. Decisions should be personalized and ideally made in collaboration with the oncology team.

### 3.6 What routes and doses of MHT are recommended?

Transdermal estradiol is the preferred method of MHT due to its lower risk of thromboembolic and metabolic side effects compared to oral formulations.^7^, ^30^, ^32^ Typical doses are in the range of 25–100 μg/day and should be tailored based on symptom severity and clinical response.^7^ The risk of stroke is unlikely to increase with transdermal estrogen at standard therapeutic doses, while oral estrogen at 0.25–1 mg/day is associated with a higher risk of stroke, especially at higher doses.^14^ Similarly, the risk of venous thromboembolism is not elevated with transdermal MHT but is increased with oral formulations.^14^

Women with an intact uterus need proper endometrial protection when using systemic estrogen therapy. Combined estrogen‐progesterone therapy with 0.045 mg/day estradiol and 0.015 mg/day levonorgestrel can be administered weekly or every 3 days using different doses and is generally well tolerated. Micronized progesterone (100–200 mg nightly) is commonly prescribed and usually well tolerated. However, the NICE 2024 guideline update found there is not enough evidence to determine whether micronized progesterone or dydrogesterone offers a lower risk of breast cancer compared with other progestogens.^14^ Still, other progestogen options can also be considered, including levonorgestrel‐releasing intrauterine systems, oral progestogens, or vaginal progesterone formulations, depending on patient preferences, tolerability, and clinical context. These options enable personalized endometrial protection while preserving the benefits of estrogen therapy.^7^, ^30^ Estrogen‐only regimens are suitable for women who have had a hysterectomy.^7^

Evidence supporting the use of tibolone in gynecologic cancer survivors remains very limited, and its oncologic safety is uncertain; therefore, most expert reviews and consensus statements recommend traditional estrogen‐based menopausal hormone therapy regimens instead of tibolone in this population.^48^

Treatment should adopt a “start low and titrate as needed” strategy, with regular reassessment of effectiveness and safety, especially in cancer survivors.^7^ When MHT is appropriate for gynecologic cancer survivors, it should follow the same core principles used in the general menopausal population, tailored to the oncologic context. Table 3 outlines the recommended formulations, dosages, routes of administration, and key prescribing considerations for this group.

**TABLE 3:** Recommended routes, formulations, and principles of MHT prescription for gynecologic cancer survivors.^a^

Parameter	Recommendation	Rationale/comments
Preferred route	Transdermal estradiol (patch or gel)	Lower thromboembolic and metabolic risk compared to oral estrogen; avoids hepatic first‐pass effect
Starting dose	Low dose: 25–37.5 μg/day (patch) or equivalent gel	“Start low and titrate as needed” approach; adjust based on symptom control and tolerability
Maintenance dose range	25–100 μg/day transdermal estradiol	Individualize according to symptom severity and clinical response
Progestogen—preferred	Micronized progesterone 100–200 mg/night (oral)	Generally well tolerated; required for endometrial protection in women with intact uterus. NICE 2024 found insufficient evidence to determine whether MP or dydrogesterone provides a lower breast cancer risk than other progestogens
Alternative progestogens	LNG‐IUS; oral progestogens; vaginal progesterone	Individualize based on preference, tolerability, and clinical context
Women post‐hysterectomy	Estrogen‐only regimen	No progestogen required; simplifies treatment
Tibolone	Not recommended in gynecologic cancer survivors	Insufficient oncologic safety data in this population; most guidelines advise against its use
Duration	Until average age of natural menopause (~51 years) in premature/early menopause	After age 51, reassess annually; benefits and risks should be reviewed regularly
Reassessment	At least annually	Evaluate symptom control, tolerability, oncologic status, and patient preferences
Local vaginal estrogen	Ultra‐low‐dose; acceptable even when systemic MHT is contraindicated	Minimal systemic absorption; first‐line for isolated GSM
Decision‐making framework	Individualized; MDT involvement when appropriate	Oncologist, gynecologist, and endocrinologist collaboration recommended; SDM with patient
Timing of initiation	After completion of primary cancer treatment and absence of active disease	No universal consensus on exact timing; most evidence supports initiation after treatment completion

Abbreviations: GSM, genitourinary syndrome of menopause; LNG‐IUS, levonorgestrel‐releasing intrauterine system; MDT, multidisciplinary team; MHT, menopausal hormone therapy; MP, micronized progesterone; NICE, National Institute for Health and Care Excellence; SDM, shared decision‐making.

^a^ This table outlines the recommended formulations, dosages, routes of administration, and key prescribing principles for MHT in gynecologic cancer survivors. Treatment should follow the same core principles used in the general menopausal population, tailored to the oncologic context. All decisions should be individualized and based on tumor histology, hormonal sensitivity, symptom severity, and patient preferences. The “start low and titrate as needed” approach is recommended, with regular reassessment of efficacy and safety.

*Source*: Hickey et al., *Lancet* 2024;^7^ Crandall et al., *JAMA* 2023;^30^ Rees et al., *Maturitas* 2020;^32^ The NAMS Advisory Panel, *Menopause* 2022;^50^ NICE, NG23 2024.^14^

#### 3.6.1 Practice recommendation

Use the lowest effective transdermal estrogen dose, combined with micronized progesterone when indicated. Begin with a low dose, gradually increase, and regularly reassess for safety and effectiveness.

### 3.7 What are safe alternatives when MHT is contraindicated?

When MHT is contraindicated, non‐hormonal strategies become essential, especially for women with hormone‐dependent cancers.^7^ If systemic MHT cannot be used due to contraindications, refusal, or cautious use, a variety of non‐hormonal options should be available within a multidisciplinary approach.

Table 4 outlines the main non‐hormonal options for managing vasomotor symptoms, including their key indications, estimated effectiveness, and level of supporting evidence. The management of GSM, including the role of local vaginal estrogen therapy and non‐hormonal vaginal treatments, is covered separately in the Section 3.8.

**TABLE 4:** Non‐hormonal pharmacological and non‐pharmacological options for managing menopausal symptoms in gynecologic cancer survivors when MHT is contraindicated or declined.^a^

Treatment category	Agent/Intervention	Primary target symptom(s)	Estimated efficacy	LoE (OCEBM)	Remarks
Pharmacological—central	Venlafaxine (SNRI)	VMS	~50%–60% reduction in hot flush frequency	1	First‐line non‐hormonal option; also improves mood and sleep
	Escitalopram/paroxetine (SSRI)	VMS	~40%–50% reduction	1	Paroxetine: avoid with tamoxifen (CYP2D6 inhibition)
	Gabapentin/pregabalin	VMS, sleep	~45%–55% reduction; especially nocturnal symptoms	2	Useful for sleep disruption; sedation may limit tolerability
	Clonidine	VMS	Modest benefit (~25%–30%)	2	Limited efficacy; side effects may restrict use
	Oxybutynin	VMS	~70% reduction in some RCTs	2	Anticholinergic effects; useful in selected patients
Pharmacological—targeted	NK3R antagonists (e.g., fezolinetant)	VMS, sleep	Significant reduction in moderate‐to‐severe hot flushes	1	Does not stimulate breast or endometrial tissue; promising for cancer survivors; long‐term oncologic safety not fully established
Local vaginal therapy	Ultra‐low‐dose vaginal estrogen	GSM (dryness, dyspareunia)	Effective for local symptoms	2	Minimal systemic absorption; generally acceptable even when systemic MHT is contraindicated; individualized MDT discussion recommended
	Vaginal lubricants/moisturizers (HA, lactic acid, Lactobacilli)	GSM	Moderate symptomatic relief	2	First‐line; safe for long‐term use; readily available
Energy‐based therapies	Fractional CO_2_ laser/Er:YAG laser	GSM	Emerging evidence	3	Limited data in cancer survivors; NICE 2024 advises against use outside controlled trials; use cautiously after RT
Psychological/behavioral	CBT (menopause‐specific)	VMS, psychological, sleep	Moderate‐to‐good improvement	1	Recommended as part of multimodal care; addresses fear of recurrence and mood; scalable delivery formats available
	Mindfulness‐based interventions	Psychological distress, sleep	Moderate benefit	2	Improves coping and QoL
	Pelvic floor physiotherapy	GSM, sexual dysfunction, urinary symptoms	Good for local symptoms	2	Essential component of survivorship care
	Psychosexual counseling	Sexual dysfunction	Effective for desire and arousal issues	2	Partner involvement recommended
Bone health	Bisphosphonates (e.g., alendronate, zoledronic acid)	Osteopenia/osteoporosis	Reduces fracture risk	1	Indicated based on DXA and FRAX; important when MHT is contraindicated
	Calcium + vitamin D supplementation	Bone health	Supportive	1	Recommended as adjunct in all patients with premature menopause

Abbreviations: CBT, cognitive behavioral therapy; CYP2D6, cytochrome P450 2D6; DXA, dual‐energy X‐ray absorptiometry; FRAX, Fracture Risk Assessment Tool; GSM, genitourinary syndrome of menopause; HA, hyaluronic acid; LoE, level of evidence; MDT, multidisciplinary team; MHT, menopausal hormone therapy; NICE, National Institute for Health and Care Excellence; NK3R, neurokinin‐3 receptor; OCEBM, Oxford Centre for Evidence‐Based Medicine; QoL, quality of life; RCT, randomized controlled trial; RT, radiotherapy; SNRI, serotonin‐norepinephrine reuptake inhibitor; SSRI, selective serotonin reuptake inhibitor; VMS, vasomotor symptoms.

^a^ This table summarizes the main non‐hormonal pharmacological and non‐pharmacological options for managing menopausal symptoms in gynecologic cancer survivors when systemic MHT is contraindicated or declined. The management of GSM, including local vaginal estrogen therapy and non‐hormonal vaginal treatments, is included. Efficacy estimates are approximate and derived from RCTs and systematic reviews in general menopausal populations; cancer‐specific data are limited for most agents. Level of evidence is graded according to the OCEBM system. CBT adapted for menopause is recommended as a first‐line behavioral intervention and can be combined with pharmacological treatments. Energy‐based therapies should be reserved for research settings pending robust long‐term efficacy and safety data.

*Source*: Crandall et al., *JAMA* 2023;^30^ Hickey et al., *Lancet* 2024;^7^ Santoro et al., *Menopause* 2020;^72^ Meczekalski et al., *J Clin Med* 2023;^23^ NICE, NG23 2024.^14^

The decline in circulating estrogen levels after menopause affects hypothalamic thermoregulation via the kisspeptin, neurokinin B, dynorphin (KNDy) neuronal network, resulting in increased neurokinin B signaling, a narrower thermoneutral zone, and vasomotor symptoms. This mechanism has facilitated the development of targeted non‐hormonal therapies. Pharmacological options include:

- SSRIs/SNRIs (e.g., venlafaxine, escitalopram, paroxetine) achieve a reduction in vasomotor symptom frequency of approximately 40%–60%.^7^, ^30^
- Gabapentinoids (gabapentin or pregabalin), especially effective for nocturnal symptoms.^7^
- Clonidine or oxybutynin, with modest benefits in selected cases.^7^
- Neurokinin‐3 receptor antagonists (e.g., fezolinetant), which directly target KNDy neurons and have shown clinically meaningful reductions in moderate‐to‐severe vasomotor symptoms and improve sleep and quality of life without stimulating breast or endometrial tissue.^30^, ^72^ It is important to periodically monitor liver function, as the drug may cause liver injury.

Although neurokinin‐3 receptor antagonists are a promising option, their long‐term safety in cancer survivors has not yet been systematically proven and their use should be tailored and monitored by specialists.^7^ Menopause‐specific CBT should be considered a formal part of multimodal symptom management for cancer survivors experiencing vasomotor symptoms, sleep disturbances, and low mood.^14^ CBT is especially beneficial in this group because it has no systemic side effects, does not interfere with cancer treatments, and can be delivered through scalable formats, such as online, group‐based, or guided self‐help options.^22^ Mindfulness‐based interventions may also improve coping skills and quality of life. Combining these behavioral strategies with pharmacological treatments might further improve symptom management and adherence to treatment.^7^ In women with osteopenia or osteoporosis, bone‐specific therapies (e.g., bisphosphonates) should be started based on fracture risk and current guidelines.

#### 3.7.1 Practice recommendation

When systemic MHT is either contraindicated or declined, non‐hormonal options like SSRIs/SNRIs, gabapentinoids, and neurokinin‐3 receptor antagonists should be offered first within a multidisciplinary care setting to address vasomotor symptoms. Management of GSM, including local vaginal estrogen, is covered in in the Section 3.8.

### 3.8 How should genitourinary symptoms be managed?

GSM covers a range of urogenital symptoms, such as vaginal dryness, dyspareunia, and urinary issues, which are common among cancer survivors and greatly affect sexual health and quality of life.^7^ Nonetheless, its definition and clinical limits are still debated, since GSM does not have universally agreed‐upon diagnostic criteria, and not all related symptoms can be solely attributed to menopause.

Vaginal dryness is a common and well‐understood symptom that often persists or worsens if untreated. The initial treatment approach should focus on non‐hormonal options, such as vaginal lubricants and moisturizers available in gel, cream, or suppository forms. These products often contain hyaluronic acid, lactic acid, and/or Lactobacilli.^7^, ^14^ They are safe, accessible, and suitable for long‐term use, especially for women who prefer to avoid hormonal therapy or have contraindications.

Local vaginal estrogen therapy is the most effective drug treatment for isolated vulvovaginal symptoms, reducing dryness, dyspareunia, and superficial dysuria with minimal systemic absorption.^14^, ^30^ It is generally safe for most gynecologic cancer survivors, particularly when MHT is unsuitable.^7^ Use should be daily, with intermittent use depending on symptoms. Importantly, systemic MHT should not be used solely for urogenital symptoms, as this may increase the risk of urinary incontinence, including stress, urgency, and mixed types.^73^, ^74^, ^75^ Pelvic organ prolapse should be evaluated via pelvic examination and confirmed with vaginal ultrasound. Physiotherapy and Kegel exercises help improve urinary symptoms. In severe cases, a pessary can be used until surgery is needed.

A core concept in GSM management is its progressive pathophysiology. Extended estrogen deprivation causes a gradual decrease in estrogen receptor expression in vulvovaginal tissues, diminishing therapy responsiveness over time. This highlights a crucial “window of opportunity,” during which early implementation of local treatments, whether hormonal or non‐hormonal, can help preserve tissue health and improve clinical outcomes.^31^, ^76^ Postponing treatment may lead to more severe atrophic changes, which could become partly irreversible.

In women with persistent symptoms, ultra‐low‐dose vaginal estrogen therapy may be considered even when systemic MHT is contraindicated, following individualized assessment and, when appropriate, multidisciplinary discussion with the oncology team.^7^, ^30^, ^32^ Beyond vaginal estrogen, additional pharmacological options include vaginal prasterone (DHEA) and oral ospemifene. Prasterone may be considered when non‐hormonal treatments or vaginal estrogen are ineffective or unsuitable, while ospemifene, an oral selective estrogen receptor modulator (SERM), may be useful in patients unable to tolerate or access local therapies, such as those with post‐radiation vaginal stenosis.^14^ However, robust safety data in gynecologic cancer survivors remain limited.

Urinary symptoms during menopause result from multiple factors, and existing evidence does not clearly link all complaints solely to estrogen deficiency. Although systemic MHT might aggravate urinary incontinence, localized vaginal estrogen treatment has been effective in alleviating dysuria, urinary frequency, urgency, and recurrent urinary tract infections (UTIs) in menopausal women.^73^, ^75^

Energy‐based therapies, such as fractional CO_2_ and Er:YAG lasers, have demonstrated promising initial results. However, existing evidence is still limited. The NICE 2024 guidelines advise against offering these treatments outside of controlled trials because of limited data on their effectiveness, possible risks like scarring, and missing cost‐effectiveness evidence.^14^ For cancer survivors, especially those who have undergone radiotherapy, a careful approach is recommended due to the risk of impaired tissue healing.

#### 3.8.1 Practice recommendation

Prioritize early use of non‐hormonal vaginal therapies for GSM; consider local estrogen or other medications based on individual assessment, and reserve energy‐based therapies for research purposes, with multidisciplinary evaluation and ongoing monitoring.

### 3.9 How can sexual dysfunction be addressed?

Sexual dysfunction after cancer is caused by multiple factors, including sudden hormonal changes, psychological stress, concerns about body image, relationship issues, and direct physical effects of cancer treatment. Common symptoms include decreased desire, arousal problems, dyspareunia, and reduced sexual satisfaction.^7^

Management should be comprehensive and tailored, incorporating education, open communication, and non‐hormonal options. Vaginal moisturizers and lubricants, pelvic floor physiotherapy, and proper management of genitourinary syndrome of menopause are essential parts of care.^7^ Psychosexual counseling and CBT strategies should be offered when available, as they can enhance coping, intimacy, and overall sexual well‐being. Partner involvement should be promoted whenever appropriate.^7^ Sexual health assessments and support should be integrated into routine survivorship care instead of being addressed only when symptoms are severe.^7^

#### 3.9.1 Practice recommendation

Provide psychosexual counseling, promote open discussion, and include pelvic floor physiotherapy and non‐hormonal treatments as part of standard survivorship care. Encourage partner involvement when appropriate.

### 3.10 What are the psychological aspects of post‐cancer menopause?

Psychological distress is common after cancer and often worsens menopausal symptoms. Fear of cancer recurrence, anxiety, depression, and concerns about health, body image, and sexuality frequently co‐exist with vasomotor symptoms and sleep issues, further lowering quality of life.^7^ Women diagnosed at a younger age and those experiencing sudden treatment‐induced menopause are especially vulnerable to psychological distress. These factors are linked to higher rates of ongoing anxiety, mood disorders, and poor coping, highlighting the importance of proactive screening and support in survivorship care.^7^

Psychological assessment should be systematically included in survivorship follow‐up care. Validated screening tools, such as the Patient Health Questionnaire‐2 or ‐9 (PHQ‐2/PHQ‐9) for depression and the Generalized Anxiety Disorder‐7 (GAD‐7) for anxiety, help identify clinically significant distress early and enable timely referral to mental health services as needed.

Clinicians should systematically distinguish between depressive symptoms related to the menopausal transition and clinical depression that meets ICD‐11 or DSM‐5 diagnostic criteria. For depressive symptoms co‐occurring with vasomotor symptoms, CBT and/or MHT may be considered.^14^ For women who meet criteria for clinical depression, treatment should follow established guidelines for depression alongside menopause‐specific care.^21^ Menopause‐specific CBT is recommended as a first‐line option for menopause‐related mood symptoms and insomnia in this population.

#### 3.10.1 Practice recommendation

Systematic mental health screening using validated tools and access to psychosocial support should be incorporated into all cancer survivorship care pathways, recognizing the increased risk of anxiety and depression after cancer.

### 3.11 What are the challenges in low‐ and middle‐income countries?

The availability of oncology and menopause care in LMICs is constrained by structural, economic, and sociocultural barriers that worsen health disparities among cancer survivors.^7^, ^11^, ^77^ Limited healthcare infrastructure, shortages of trained professionals, financial limitations, low health literacy, and geographic obstacles hinder access to diagnosis, treatment, and survivorship services. Cancer care is mostly focused in urban areas, leading to delays in diagnosis and fragmented follow‐up for rural populations. Persistently low health spending in many regions restricts access to essential medicines, supportive therapies, and comprehensive survivorship programs.^11^, ^78^ Women in these settings are often diagnosed at younger ages and face a disproportionate burden of treatment‐related menopause. However, survivorship care often emphasizes cancer recurrence over long‐term symptom management, resulting in under‐recognition and undertreatment of menopausal and psychosocial symptoms, which negatively affect quality of life.^7^, ^77^

Recent multinational data highlight the extent of these disparities. A pharmacist survey across six LMICs showed an overall MHT product availability of only 68.9%, ranging from 92.7% in Nepal to 42% in Nigeria, with notable differences between urban and rural areas.^79^ Even when available, high out‐of‐pocket costs, supply shortages, and limited provider confidence significantly hinder access. In India, although 48% of women say they would use MHT if offered, actual prescription rates by primary care providers are below 1%.^80^ These barriers to access are especially severe for gynecologic cancer survivors in LMICs, who may face the added challenge of younger age at diagnosis, more aggressive treatment‐induced menopause, and fewer resources for survivorship care.

#### 3.11.1 Practice recommendation

Develop community‐based and telehealth programs that integrate menopause and cancer follow‐up care, using primary healthcare infrastructure to lessen disparities in survivorship care.

### 3.12 What is the role of multidisciplinary care?

Multidisciplinary care is crucial for effective survivorship management after cancer, facilitating coordinated assessment and treatment of physical, psychological, and social impacts of cancer and its therapies.^7^, ^81^ Survivorship care should involve oncologists, gynecologists, endocrinologists, psychologists, and allied health professionals to ensure comprehensive management of menopausal symptoms, sexual health, mental well‐being, and long‐term health issues. This approach promotes early identification of unmet needs, shared decision‐making, and ongoing care throughout survivorship.^7^, ^81^ Coordinated multidisciplinary models are associated with better symptom control, higher quality of life, and increased patient satisfaction, and are especially vital for managing menopause after cancer, where fragmented care can lead to underdiagnosis and undertreatment.^7^ Effective survivorship care for gynecologic cancer survivors requires a structured, multidisciplinary, and long‐term strategy that addresses menopausal health alongside oncologic follow‐up. Table 5 details the key domains, recommended assessments, validated tools, and review intervals to inform clinical practice.

**TABLE 5:** Essential elements of a structured multidisciplinary survivorship care model for gynecologic cancer survivors undergoing treatment‐induced menopause.^a^

Care domain	Assessment/Intervention	Validated tool/Method	Minimum review interval	Responsible professional(s)
Menopausal symptoms	Systematic symptom evaluation	MRS; MENQOL	Every visit; at least every 6–12 months	Gynecologist; menopause specialist
Bone health	DXA scan; fracture risk assessment	DXA (FRAX score)	Baseline; then every 1–5 years based on risk	Gynecologist; endocrinologist; rheumatologist
Cardiovascular health	BP, lipid profile, weight, lifestyle	Framingham/WHO CVD risk tools	Annually	PCP; cardiologist (if indicated)
Psychological well‐being	Depression and anxiety screening	PHQ‐9; GAD‐7; IES‐R	Every visit; at least annually	Psychologist; psychiatrist; PCP
Sexual health	Sexual function assessment; GSM evaluation	FSFI; PISQ‐12	Every visit; at least annually	Gynecologist; psychosexual counselor
Fertility and ovarian function	Ovarian reserve; menstrual recovery monitoring (premenopausal patients)	FSH, AMH, AFC (when applicable)	Individualized; 6–12 monthly in women aged <40	Reproductive endocrinologist; oncologist
Oncologic surveillance	Disease recurrence monitoring	Cancer‐specific imaging and biomarkers per guidelines	Per tumor‐specific protocol	Gynecologic oncologist
Genitourinary symptoms	Pelvic floor assessment; GSM management	Clinical evaluation; POP‐Q; validated questionnaires	At least annually	Gynecologist; pelvic floor physiotherapist
Lifestyle and preventive care	Physical activity, nutrition, smoking cessation, weight management	Clinical counseling; structured survivorship care plan	Annually	MDT; PCP
Treatment review	MHT or non‐hormonal therapy reassessment	Clinical assessment; symptom scores	At least annually	Gynecologist; menopause specialist
Access and equity	Identification of barriers to care (geographic, economic, cultural)	Social assessment; patient‐reported barriers	At each care transition	Social worker; PCP; telehealth services

Abbreviations: AFC, antral follicle count; AMH, anti‐Müllerian hormone; BP, blood pressure; CVD, cardiovascular disease; DXA, dual‐energy X‐ray absorptiometry; FRAX, Fracture Risk Assessment Tool; FSFI, Female Sexual Function Index; FSH, follicle‐stimulating hormone; GAD‐7, Generalized Anxiety Disorder‐7; GSM, genitourinary syndrome of menopause; IES‐R, Impact of Event Scale–Revised; MDT, multidisciplinary team; MENQOL, Menopause‐Specific Quality of Life Questionnaire; MHT, menopausal hormone therapy; MRS, Menopause Rating Scale; PCP, primary care physician; PHQ‐9, Patient Health Questionnaire‐9; PISQ‐12, Pelvic Organ Prolapse/Urinary Incontinence Sexual Questionnaire‐12; POP‐Q, Pelvic Organ Prolapse Quantification System; WHO, World Health Organization.

^a^ This table outlines the key domains, recommended assessments, validated tools, and minimum review intervals for a structured, multidisciplinary survivorship care model addressing treatment‐induced menopause in gynecologic cancer survivors. The model is designed to be adaptable to local healthcare resources and can be implemented across high‐income and low‐ and middle‐income settings using resource‐stratified approaches. All assessments should be integrated into routine oncologic follow‐up and survivorship care planning.

*Source*: Hickey et al., *Lancet* 2024;^7^ Emery et al., *Lancet* 2022;^81^ Woopen et al., *Cancer Treat Rev*. 2022;^4^ NCCN Survivorship Guidelines 2026;^5^ Slart et al., *Eur J Nucl Med Mol Imaging* 2025.^25^

#### 3.12.1 Practice recommendation

Establish structured, multidisciplinary survivorship services that address menopause, sexual health, and mental well‐being after cancer.

### 3.13 How should long‐term follow‐up of menopausal symptoms be structured?

Long‐term follow‐up of menopausal symptoms in cancer survivors should be structured, multidisciplinary, and personalized, with symptom and treatment reviews conducted at least annually or sooner if clinical changes occur.^7^ Follow‐up should include a systematic assessment of vasomotor, genitourinary, sexual, sleep, and psychological symptoms, utilizing clinical history and patient‐reported outcomes to inform management. Treatment plans should be regularly evaluated and adjusted based on symptom severity, cancer type, ongoing therapies, and patient preferences.^7^, ^81^

Surveillance for late effects and comorbidities should be incorporated into follow‐up care, including evaluating bone health, cardiovascular risk, and psychosocial well‐being, in coordination with oncology and primary care services.^7^, ^27^ Multidisciplinary teamwork and shared survivorship care plans are vital to maintain continuity of care, especially during the transition from oncology to long‐term follow‐up. Telemedicine can be used to support symptom monitoring and improve access to care when appropriate.^7^

#### 3.13.1 Practice recommendation

Ensure continuity of survivorship care by regularly reassessing menopausal symptoms and changing cardiovascular, bone, and emotional health needs, using a life‐course approach.

## 4 FINAL CONSIDERATIONS

Menopause after cancer treatment is an increasingly recognized aspect of cancer survivorship. As oncologic outcomes improve worldwide, more women are living long enough to face the long‐term hormonal effects of cancer therapy, including treatment‐induced menopause and its related physical, psychological, and sexual health impacts.

Managing menopausal symptoms in cancer survivors requires balancing oncologic safety with quality of life. Although MHT is the most effective for vasomotor and genitourinary symptoms, it should be personalized based on cancer type, tumor biology, and patient preferences. Evidence indicates that systemic MHT might be suitable for some non–hormone dependent gynecologic cancer survivors, but non‐hormonal options remain crucial for women with hormone‐sensitive tumors or hormone therapy contraindications. Local vaginal estrogen therapy is generally safe for most gynecologic cancer survivors and can effectively treat genitourinary syndrome of menopause when systemic treatment is not appropriate.

Importantly, menopause after cancer should not be seen only as a symptom‐management issue but as a larger survivorship challenge that needs long‐term multidisciplinary care. Focusing on bone health, cardiovascular risk, sexual function, and psychological well‐being is crucial to improving long‐term outcomes and quality of life for cancer survivors.

Significant disparities continue in access to survivorship and menopause care worldwide, especially in low‐ and middle‐income countries where gynecologic cancers are more common and health systems face structural challenges. Addressing these gaps is essential to improving women's health globally. Incorporating menopause care into survivorship programs, enhancing multidisciplinary collaboration, and increasing access to safe and effective treatments are crucial priorities for healthcare systems.

To promote consistent and high‐quality care for gynecologic cancer survivors across different healthcare settings, we recommend a structured checklist for managing menopause (Box 1). This tool aims to help clinicians navigate four key areas: pre‐treatment counseling, early survivorship assessment, ongoing follow‐up, resource‐efficient care for LMICs, and patient‐centered communication. The checklist is intended to support, not replace, personalized clinical judgment and team‐based decisions. It can be adapted to local protocols, available resources, and patient preferences.

BOX 1

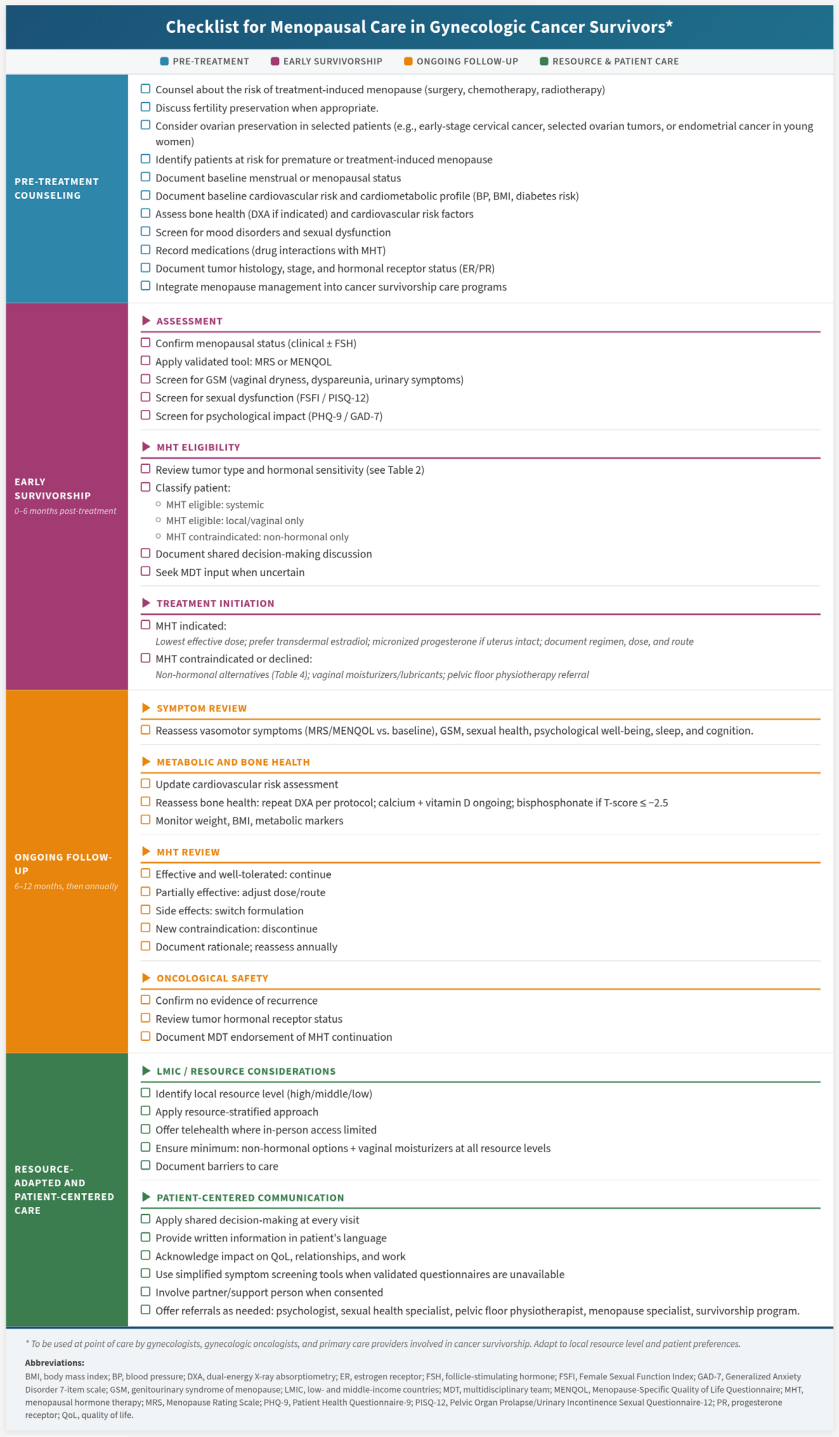

*To be used at point of care by gynecologists, gynecologic oncologists, and primary care providers involved in cancer survivorship. Adapt to local resource level and patient preferences.

Abbreviations: BMI, body mass index; BP, blood pressure; CBT, cognitive behavioral therapy; CVD, cardiovascular disease; DXA, dual‐energy X‐ray absorptiometry; ER, estrogen receptor; FSFI, Female Sexual Function Index; FSH, follicle‐stimulating hormone; GAD‐7, Generalized Anxiety Disorder‐7; GSM, genitourinary syndrome of menopause; LMIC, low‐ and middle‐income country; MDT, multidisciplinary team; MENQOL, Menopause‐Specific Quality of Life Questionnaire; MHT, menopausal hormone therapy; MP, micronized progesterone; MRS, Menopause Rating Scale; PHQ‐9, Patient Health Questionnaire‐9; PISQ‐12, Pelvic Organ Prolapse/Urinary Incontinence Sexual Questionnaire‐12; PR, progesterone receptor; QoL, quality of life; RT, radiotherapy; SDM, shared decision‐making; VMS, vasomotor symptoms.

In this context, the FIGO Committee on Women at Menopausal Age emphasizes the importance of a life‐course approach to women's health after cancer. Clinicians should implement personalized, evidence‐based strategies that balance oncologic safety with comprehensive symptom management and long‐term preventive care. Enhancing survivorship care for women after cancer is vital not only for improving quality of life but also for ensuring that the growing population of cancer survivors receives fair, holistic, and patient‐centered care worldwide. As the number of cancer survivors continues to increase globally, integrating menopause care into oncologic survivorship programs is a crucial step toward improving long‐term health and quality of life for women everywhere. Ultimately, decisions about managing menopause after cancer should prioritize patient autonomy and shared, informed choices. Women should receive thorough, unbiased information on the advantages, risks, and uncertainties of all options, including MHT, non‐hormonal therapies, and behavioral interventions, so they can select options that match their values, preferences, and individual clinical situations.

## AUTHOR CONTRIBUTIONS

**Agnaldo Lopes da Silva‐Filho**: Conceptualization, literature review, methodology design, original draft preparation, and coordination of revisions. **Suvarna Khadilkar**: Supervision as Committee Chair, critical revision, and expert review of the manuscript. **Hema Divakar**: Critical revision and expert review of the manuscript. **Chiara Benedetto**: Contribution to specific sections, critical review, and approval of the final version. **Andrea R. Genazzani**: Contribution to specific sections, critical review, and final approval. **Diana Ramos**: Contribution to specific sections, critical review, and final approval. **Elizabete Argale**: Contribution to specific sections, critical review, and final approval. **Enrique Herrera**: Contribution to specific sections, critical review, and final approval. **Martha Hickey**: Contribution to specific sections, critical review, and final approval. All authors meet ICMJE criteria for authorship and have read and approved the final manuscript.

## FUNDING INFORMATION

No specific funding was received for this work.

## CONFLICT OF INTEREST STATEMENT

The authors have no conflicts of interest.

## DISCLOSURE OF AI‐ASSISTED TECHNOLOGIES IN THE WRITING PROCESS

While preparing this manuscript, the authors utilized AI‐assisted language editing tools for tasks such as language editing, organizing literature, or drafting support. They reviewed and revised all AI‐produced material and assume full responsibility for the final published content.

## Supporting information


**Table S1.** Structured menopause consultation template for cancer survivors.

## Data Availability

Data sharing is not applicable to this article as no new data were created or analyzed in this study. This manuscript is a narrative review and position paper based on previously published literature.
