# Supplementary material for: Menopausal hormone therapy and comprehensive postmenopausal care in gynecologic cancer survivors: A position paper from the FIGO Committee on Women at Menopausal Age
Source: Int J Gynaecol Obstet. 2026 Jul 22;174(3):1151–71. doi: 10.1002/ijgo.71188 (PMC13505246; doi:10.1002/ijgo.71188)
Supplement: Supplementary file 1 [file IJGO-174-1151-s001.docx]

**Supplementary Table 1.** Structured menopause consultation template for cancer survivors.

| Menopause workup | Symptom | Severity (1–4) | Yes/No |
| --- | --- | --- | --- |
| Hot flushes/sweating | Heat/cold temperature |  |  |
|  | Skin problem/itching |  |  |
| Heart discomfort | Palpitation/respiratory |  |  |
| Sleep difficulty | Ability to stay asleep |  |  |
|  | Onset of sleep |  |  |
|  | Length of sleep |  |  |
| Depression/anxiety | Sad/not functioning well |  |  |
|  | Nervous/restless |  |  |
| Physical/mental fatigue | Tired |  |  |
|  | Attention decreased |  |  |
|  | Memory lapses |  |  |
| Sexual function change | Decreased |  |  |
| Bladder function | Difficulty urinating |  |  |
|  | Urine frequency |  |  |
|  | Urine loss/retention |  |  |
| Vaginal dryness | Irritation/burning |  |  |
|  | Pain having sex |  |  |
| Joint/muscle | Limited function |  |  |
|  | Joint pain |  |  |
|  | Muscle pain |  |  |
| Bleeding | Occasional |  |  |
|  | Frequent |  |  |
| Pelvic pain | Middle |  |  |
|  | Left |  |  |
|  | Right |  |  |
| Breast | Tender/lump |  |  |
| Weight increased | Recent |  |  |

This template offers a comprehensive framework to standardize clinical evaluation and improve patient–physician communication across various menopause-related symptom areas. It aims to facilitate shared decision-making, enhance symptom recognition, and support personalized survivorship care.
